# Brain Iron Accumulation in Atypical Parkinsonian Syndromes: *in vivo* MRI Evidences for Distinctive Patterns

**DOI:** 10.3389/fneur.2019.00074

**Published:** 2019-02-12

**Authors:** Jae-Hyeok Lee, Myung-Sik Lee

**Affiliations:** ^1^Department of Neurology, Research Institute for Convergence of Biomedical Science and Technology, Pusan National University Yangsan Hospital, Pusan National University School of Medicine, Yangsan, South Korea; ^2^Department of Neurology, Gangnam Severance Hospital, Yonsei University College of Medicine, Seoul, South Korea

**Keywords:** iron, neurodegeneration, magnetic resonance imaging, atypical parkinsonian syndromes, multiple system atrophy, progressive supranuclear palsy

## Abstract

Recent data suggest mechanistic links among perturbed iron homeostasis, oxidative stress, and misfolded protein aggregation in neurodegenerative diseases. Iron overload and toxicity toward dopaminergic neurons have been established as playing a role in the pathogenesis of Parkinson's disease (PD). Brain iron accumulation has also been documented in atypical parkinsonian syndromes (APS), mainly comprising multiple system atrophy (MSA), and progressive supranuclear palsy (PSP). Iron-sensitive magnetic resonance imaging (MRI) has been applied to identify iron-related signal changes for the diagnosis and differentiation of these disorders. Topographic patterns of widespread iron deposition in deep brain nuclei have been described as differing between patients with MSA and PSP and those with PD. A disease-specific increase of iron occurs in the brain regions mainly affected by underlying disease pathologies. However, whether iron changes are a primary pathogenic factor or an epiphenomenon of neuronal degeneration has not been fully elucidated. Moreover, the clinical implications of iron-related pathology in APS remain unclear. In this review study, we collected data from qualitative and quantitative MRI studies on brain iron accumulation in APS to identify disease-related patterns and the potential role of iron-sensitive MRI.

## Introduction

Iron overload in the substantia nigra and its toxicity toward dopaminergic neurons has been suggested to play a key role in the pathogenesis of Parkinson's disease (PD) (1). Brain iron accumulation has also been repeatedly documented in atypical parkinsonian syndromes (APS), mainly comprising multiple system atrophy (MSA), and progressive supranuclear palsy (PSP). Pathological studies have shown that iron levels in the putamen, globus pallidus, and substantia nigra are higher in MSA patients than in PD and control patients, and resembles the levels found in PSP patients (2–4).

Iron-sensitive magnetic resonance imaging (MRI) has been applied to identify iron-related signal changes for the diagnosis and differentiation of these disorders. However, the clinical and pathogenic implications of iron-related pathology detected by MRI in APS remain unclear. In this review study, we collected data from qualitative and quantitative MRI studies on brain iron accumulation in APS to identify disease-related patterns and the potential role of iron-sensitive MRI.

### Potential Pathogenic Mechanisms of Iron Accumulation in APS

Brain iron accumulation can be caused by several factors, such as impaired iron homoeostasis, neuroinflammation, and increased blood–brain barrier permeability (1). The key proteins associated with degenerative parkinsonism, α-synuclein and tau, are believed to play roles in iron homeostasis (5). α-synuclein is a cellular ferrireductase responsible for reducing ferric iron (Fe^3+^) to ferrous iron (Fe^2+^) (6). In the presence of functional tau, the export of ferrous iron in neurons is controlled by the binding of the amyloid precursor protein (APP) to tau, which results in the trafficking of the protein complex to the neuronal surface, where APP interacts with ferroportin (7). Changes in these proteins might result in altered cellular iron homeostasis (5).

Dysregulation of the proteins involved in iron homeostasis has been reported in APS patients. In PSP patients, the amount of ferritin, an iron-storing protein, has been reported to be considerably lower than that in control patients, which is the opposite trend of increasing iron burden (8). High levels of iron might exceed the iron-buffering capacity of complexes, such as neuromelanin and ferritin in the substantia nigra (1, 9). In MSA, an increase in ferritin iron coupled with a reduction in ferroportin expression has been detected in the pons and putamen (10). These findings suggest a potential deficit in bioavailable iron despite an excess of tissue iron.

Impaired iron homeostasis can induce toxic protein oligomers and abnormal intracellular aggregates under pathological conditions. The aggregation of α-synuclein and tau has been shown to be triggered by iron *in vitro* (1). Iron promotes α-synuclein aggregation by inducing β-sheet conformational changes and increasing oxidative stress and microglial activation (4, 11). Abundant evidence has demonstrated the role of iron in tau hyperphosphorylation and aggregation into fibrils (12, 13).

In postmortem brain tissues, high concentrations of iron have been colocalized with α-synuclein and hyperphosphorylated tau aggregates. Notably, the highest iron content in the putamen, as assessed by histochemistry, is correlated with the highest density of glial cytoplasmic inclusions and α-synuclein aggregation in MSA brains (14). Hyperphosphorylated tau aggregates from the brains of PSP patients have been shown to colocalize with ferritin (15).

Iron overload in brain regions that undergo protein misfolding and aggregation can promote oxidative stress and neuroinflammation (4). In turn, iron-induced oxidative stress leads to apoptosis and ferroptosis, a novel mode of regulated cell death dependent on iron and lipid peroxidation (1, 16). Scavenging of accumulated labile iron by chelation can protect against iron-mediated neuronal damage (17).

## Current MRI Methods for Detection and Quantification of Brain Iron

### Iron-Sensitive MRI Techniques

Iron-sensitive MRI techniques have been used to differentiate parkinsonian disorders and monitor disease progression (18–20). Various methods have been used to study iron content, including R2 (1/T_2_), ${\text{R}}_{2}^{*}$ (1/${\text{T}}_{2}^{*}$), R′2 (1/${\text{T}}_{2}^{\prime }$), susceptibility weighted imaging (SWI), and quantitative susceptibility mapping (QSM) (20, 21). SWI enhances image contrast by using the susceptibility differences between tissues; images are obtained by combining T2^*^-weighted magnitude images with filtered phase images in the gradient echo data. SWI further enhances the contrast between tissues of different susceptibilities (22). Because of its high degree of sensitivity in detecting and visualizing iron deposition, SWI has been applied in the diagnosis and differentiation of parkinsonian disorders (23). However, SWI does not provide quantitative measurement of magnetic susceptibility, which is a limitation that has been addressed by the recent development of QSM (22, 23). Both QSM and the transverse relaxation rate (R2^*^) are highly sensitive and represent the most accurate measurement of iron content in the brain (20); results have been shown to correlate closely with iron concentrations measured in postmortem brain tissues (24, 25). QSM can also overcome the confounding effect of increased water content influencing relaxation values, thus counteracting the effect of increased iron levels (20). Recent studies have shown that QSM achieves stronger diagnostic performance than R2^*^ for detecting iron deposition in the subcortical gray matter (26, 27). However, the opposite effect of myelin on QSM may impede its positive correlation with iron. A histological validation study found lower correlation coefficients for QSM and iron in white matter and cortical gray matter regions than in the basal ganglia when correlating R2^*^ and iron (28).

### Quantitative MRI Measurements of Brain Iron

The region-of-interest (ROI) method is commonly used to calculate regional iron content. The method is based on the manual or automated segmentation of target structures (29). Automated ROI analysis eliminates bias in the process of selecting images and drawing ROIs (30). Computer-based MRI analyses using machine learning techniques and support vector machine (SVM) classification have proven potential in the discrimination of parkinsonian syndromes and the tracking of disease progression (31, 32). SVM pattern recognition of SWI data can accurately discriminate PD from APS (32).

Focal or unevenly increased iron deposition should be considered in determining the iron concentration within a structure (23, 33). Single or two consecutive ROIs in two-dimensional images without delineation of the exact boundaries of a structure may not sufficiently reflect unevenly or focally increased iron content of the whole three-dimensional structure. Voxel-based analysis can provide additional information about the topographic distribution of sub-regional iron deposition (34).

The multimodal MRI approach combines MRI techniques for imaging iron and other MRI sequences that are sensitive to complementary tissue characteristics, including volume atrophy and microstructural damage (35). This approach may aid early diagnosis, monitoring of disease progression, and highlighting of pathogenic mechanisms (36, 37).

## Iron-deposition Patterns in APS: the Determinants of Region Specificity

### Age-Related and Structure-Specific Patterns

The regional distribution of iron is heterogeneous in normal adult brains. The basal ganglia have the highest iron concentrations, whereas low concentrations are detected in the cortical gray matter, white matter, brainstem, and cerebellum (1). Typically, total iron concentrations increase with age in the substantia nigra, putamen, globus pallidus, caudate nucleus, and cortices. However, the rate of iron accumulation varies among strictures throughout the adult life span (38, 39). For example, a previous study found high iron concentration in the globus pallidus regardless of age, but a significantly greater presence of iron in the putamen with advancing age (38). Regional heterogeneity of brain iron and its changes with age have been confirmed *in vivo* by MRI (1).

The region- or structure-specific pattern of iron deposition in normal aging can be enhanced in pathologic conditions. In the parkinsonian variant of MSA (MSA-P), iron deposition predominantly in the posterolateral part of the putamen is in line with the aging pattern (40, 41). The motor-related subcortical structures may present with different patterns in both volume and iron level. In previous MRI studies, the putamen demonstrated a decrease in volume and an increase in iron level, whereas the thalamus did not show iron deposition despite volume shrinkage (42, 43).

### Pathoanatomy-Related Distribution

**Figure 1:**
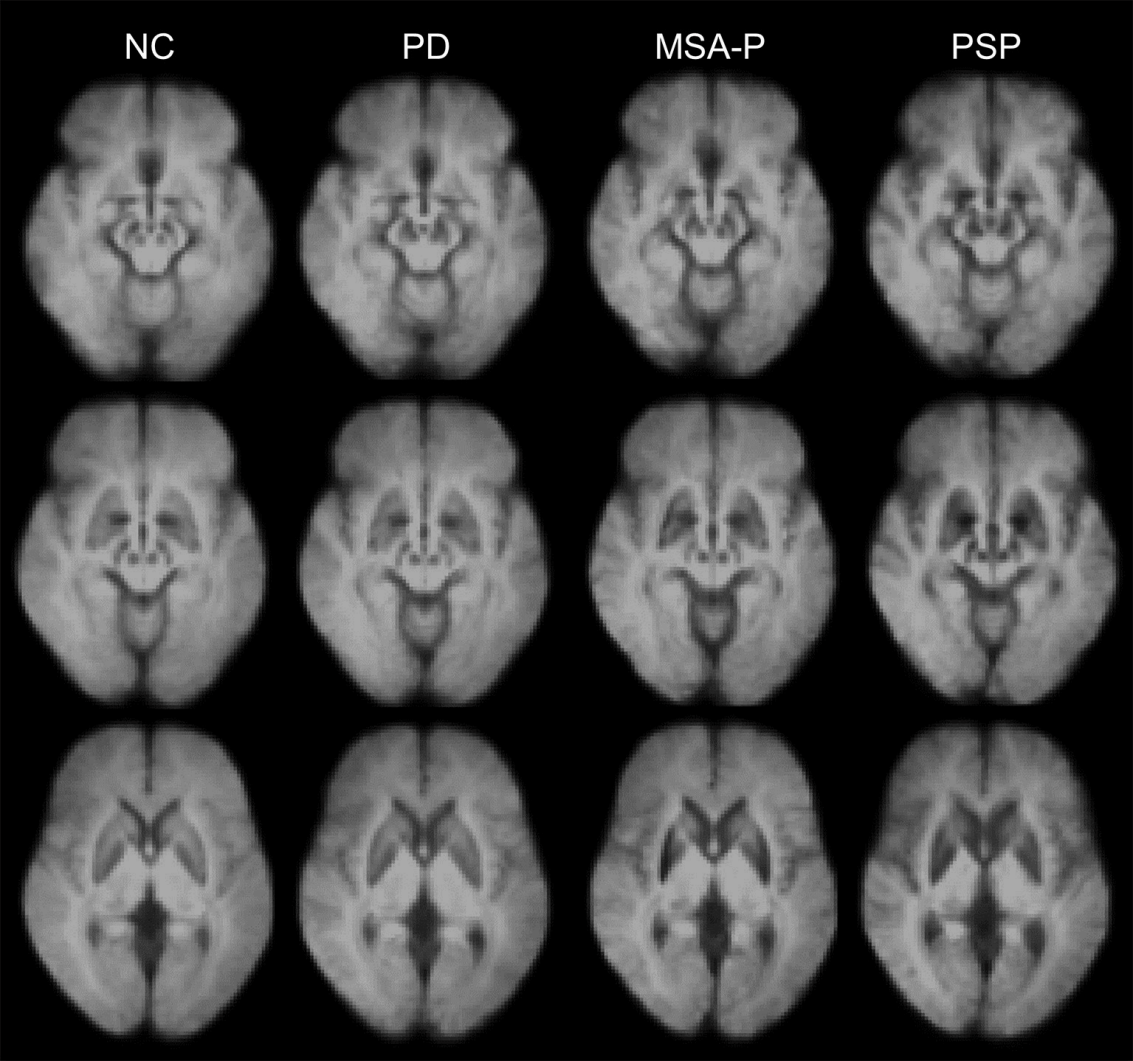
Disease-specific susceptibility weighted imaging maps generated using the mean images of normal control (NC), patients with Parkinson's disease (PD), the parkinsonian variant of multiple system atrophy (MSA-P), and progressive supranuclear palsy (PSP). The images were obtained from the Pusan National University Yangsan Hospital using protocols approved by the institutional review board. Written informed consent was obtained from all participants.

Total iron levels have been found to be elevated in most areas of the basal ganglia in the postmortem brain tissue of patients with APS (2). However, *in vivo* iron-sensitive MRI has shown topographical differences in excessive iron accumulation among the distinct subtypes of parkinsonism (23, 33, 34, 43–46). Significant increases in iron-related signals (SWI phase and QSM susceptibility) have been found in the midbrain and globus pallidus of patients with PSP, and in the putamen of patients with MSA (Figure 1) (34, 46).

Regions of higher iron content are correlated with the main sites of underlying pathology. Increased iron in the putamen is the most consistent finding in MSA (23, 33, 34, 43–47), and specifically in MSA-P (48, 49), as well as being correlated with hypometabolism on ^18^F-flurodeoxyglucose positron-emission tomography (PET) (50). In a previous study, *in vivo* ^18^F-flortaucipir tau PET revealed that PSP patients had the highest overall uptake in the globus pallidus with the greatest discriminant power (51).

The multimodal MRI approach combining volumetry and iron estimation has shown that excessive iron accumulation is associated with advanced atrophy in a region-specific manner (4, 43, 49). R2^*^ values are negatively correlated with volumes in the putamen and globus pallidus (43). In a longitudinal study, iron accumulation in the putamen increased in parallel with the extent of atrophy (49).

### Uneven or Sub-structural Distribution

Iron distribution can be uneven across the entire structure of the brain. Iron concentrations in MSA-P are highest in the posterolateral subregion of the putamen (33, 41, 44, 48). Sub-structural differences have also been demonstrated by voxel-based analysis (34, 43). A previous study reported significant increases in SWI hypointensity in the posterolateral putamen and adjacent lateral aspect of the globus pallidus in patients with MSA-P, and in the anterior and medial aspects of the globus pallidus in patients with PSP (34). Similarly, higher R2^*^ values have been reported in more atrophic sub-regions (43). In MSA-P, increases in R2^*^ values in the putamen and globus pallidus have been noted more posteriorly and dorsally than those in PSP.

Conventional MRI techniques are insufficient to delineate the inner structure of the substantia nigra and measure the loss of melanized dopaminergic neurons and the increase of iron deposition (52). Recently, iron-sensitive MRI sequences at high field strengths have been reported to consistently display a hyperintense ovoid area within the dorsolateral border of the otherwise hypointense substantia nigra pars compacta, which is referred to as dorsolateral nigral hyperintensity (53–55). A postmortem 9.4 T MRI study with histopathological correlation indicated that this MRI feature corresponds to nigrosome-1 (56). Loss of dorsolateral nigral hyperintensity, a typical finding for patients with PD, is also observed in the majority of patients with MSA or PSP (53–55).

## Clinical Implications of Brain Iron Deposition in APS

### Discriminant Markers

Distinctive patterns of iron deposition detected on iron-sensitive MRI can be used in clinical practice to differentiate PD, MSA, and PSP (see Table 1). Patients with PSP have more widespread iron deposition in the motor-related subcortical nuclei than patients with other parkinsonian disorders (Figure 1) (34, 61). In receiver operating characteristic (ROC) analyses, the putamen has been consistently demonstrated to have the highest area under the curve (AUC) values (~0.8–0.9) in differentiating MSA-p from PSP and PD (34, 45, 46, 58, 59, 63). Furthermore, the red nucleus and globus pallidus are the two most valuable nuclei in the diagnosis of PSP (34, 46). However, direct comparisons among studies are difficult because of differences in patient characteristics (e.g., clinical severity and clinical subtypes) and methodology (e.g., MRI parameters, selected brain structures, and analysis methods) (31). The role of iron-sensitive MRI in other APS, such as diffuse Lewy body disease and corticobasal degeneration, has not yet been determined.

**Table 1:** Summary of studies using iron-sensitive MRI in atypical parkinsonian syndromes.

**Study**	**References**	**Number of subjects**	**MRI methods**	**Selected structures**	**Analysis methods**	**Main findings**
Signal intensity grading	Gupta et al. (57)	12 MSA-P, 12 PSP, 11 PD, 11 NC	SWI, 1.5T	RN, SN, DN, PUT	Grade 0–3 on the basis of the mean SI values	1. Hypointensity score of SN and RN: higher in PSP than that of MSA-P and PD.2. Hypointensity score of PUT: higher in PSP than that of PD.
	Sakurai et al. (44)	10 MSA-P, 10 PD, 10 NC	PRESTO, T2*WI, 3T	PUT	PUT SI scored in comparison with that of the GP	1. PUT signal changes: the posterolateral part with a striking lateral to medial gradient.2. PRESTO: lower intensity and better than T2*WI.
	Lee and Baik (41)	11 MSA-P, 30 PD, 30 NC	SWI, 3T	PUT	Grade 0-3 on the basis of the pattern of hypointensity	The pattern of posterolateral PUT: a striking lateral to medial gradient (a grade of ≥2) differentiated MSA-P from PD and NC.
	Sugiyama et al. (58)	15 MSA, 9 PSP, 16 PD, 10 NC	T2WI, T2*WI, 1.5T	PUT	PUT abnormality scores on visual analog scale	1. PUT hypointensity on T2*WI: the highest diagnostic accuracy.2. AUCs of 0.797 (vs. PSP), 0.867 (vs. PD), and 0.896 (vs. NC) for differentiating MSA from PSP, PD and NC.
	Wang et al. (59)	18 MSA-P, 21 MSA-C, 18 PD, 31 NC	SWI, 3T	PUT, SN	Grade 0-3 on the basis of the mean SI values of PUT; “Swallow-tail” sign (nigrosome 1) of SN	AUC of combined signs: increased from 0.85 (swallow tail) or 0.68 (PUT hypointensity) to 0.93.
ROI-based quantification of iron	Wang et al. (33)	8 MSA-P, 16 PD, 44 NC	SWI, 1.5T	SN, RN, CN, PUT, GP, TH (PT)	Manually-drawn ROI (2D); Average-total-iron-deposition values (phase shift) and high-iron-content area percentages; 4 subregions of the PUT	1. The high-iron-deposition-percentage area: superior to the average phase shift in differentiating MSA-P from PD (PUT: AUC = 0.88 vs. 0.78; PT: AUC = 0.79 vs. 0.62).2. Lower inner region of the putamen: the most valuable subregion.
	Han et al. (34)	12 MSA-P, 11 PSP, 15 PD, 20 NC	SWI, 3T	RN, SN, CN, GP, PUT, TH	Group comparisons of mean phase shift values in the manually-drawn ROI (2D); Voxel-based analysis of the processed SWI	1. PUT (AUC = 0.836): the most valuable nucleus in differentiating MSA-P from PSP and PD.2. GP (AUC = 0.869) and TH (AUC = 0.884): the two most valuable nuclei in differentiating PSP from MSA-P and PD.3. Sub-regional differences in SWI hypointensity in the PUT, GP, and TH between MSA-P and PSP.
	Sakurai et al. (45)	13 MSA-P, 12 PSP, 12 PD, 13 NC	PRESTO, 1.5T	RN, SN, DN, STN, PUT	Volume of interest (VOI) analysis of normalized images; Comparison of SI ratio in target VOIs	1, PUT: the highest AUCs of 0.83 (vs. PSP) and 0.91 (vs. NC) in the diagnosis of MSA-P.2. RN: the highest AUCs of 0.87 (vs. MSA-P), 0.90 (vs. PD), and 0.89 (vs. NC) in the diagnosis of PSP.
	Sjöström et al. (46)	11 MSA, 15 PSP, 62 PD, 14 NC	QSM, 1.5/3T	SN, RN, PUT, GP	Group comparisons of susceptibility in the manually-drawn ROI (2D)	1. RN: the most promising biomarker for separating groups, especially for PSP (AUC of 0.97 for PSP vs. PD, 0.86 for MSA vs. PD and 0.75 for PSP vs. MSA).2. GP: a similar accuracy in separating PSP from MSA of 0.73.
	Yoon et al. (50)	17 MSA-P, 30 PD	SWI, 3T	PUT	Group comparisons of mean SI values of the anterior and posterior halves of the PUT in the manually-drawn ROI; Correlation of the ROI SI values of SWI and SUVR on 18F-FDG PET	1. The values of dominant-side of the posterior half of the PUT: high AUC values (AUC of 18F-FDG PET = 1; AUC of SWI = 0.947) to differentiate MSA-P and PD.2. The low SI in the putamen on SWI correlated with hypometabolism on 18F-FDG PET in MSA-P.
	Hwang et al. (60)	27 MSA-P, 50 PD, 27 NC	SWI, 3T	PUT	Quantitatively measured PUT width and phase-shift values	1. Significantly higher asymmetric phase-shift value of the posterior PUT in MSA-P.2. A contralateral correlation between the symptomatic side and the marked hypointense signal side.
	Boelmans et al. (61)	12 PSP, 30 PD, 24 NC	T2, T2*, T2′, 1.5T	CN, PUT, GP, TH, WM	Group comparisons of mean T2′ values in the manually-drawn ROI; A stepwise linear discriminant analysis to predict the clinical diagnosis	1. Shortened T2′ values in the CN, PUT, and GP in PSP compared to PD and NC. 2.T2′ mean values: excellent discrimination between PSP and PD patients.
	Lee et al. (62)	24 PSP, 20 NC	R2*,3T	SN, STN, DN, PUT, GP	Correlations between R2* values and UPDRS	1. Significantly higher R2* values in all of the five brain regions in PSP patients.2. UPDRS subscores correlated with R2* values.
Multimodal MRI analysis	Focke et al. (63)	10 MSA-P, 9 PSP, 12 PD, 13 NC	R2*, R2, R1, DTI, MT, 3T	SN, CN, PUT, GP	Group comparisons of quantitative MRI data in the manually-drawn ROI (3D)	R2* mapping in the PUT: the best separation of MSA from PD patients and controls with a good predictive power (AUC of ≤ 0.96).
	Lee et al. (43)	15 MSA-P, 13 PSP, 29 PD, 22 NC	R2*, T1-Vol., 3T	CN, PUT, GP, TH	Automated ROI (3D) analysis for R2* and volume calculation; Voxel-based analysis to visualize a topographical correlation of R2* and volume	1. Negative correlation between R2* values and volumes in the PUT (*r* = −0.777, *p* < 0.001) and GP (*r* = −0.409, *p* = 0.025) of MSA-P, and GP (*r* = −0.4, *p* = 0.043) of PSP.2. Higher R2* values in more severely atrophic sub-regions.
	Lee et al. (49)	8 MSA-P, 9 MSA-C, 15 PD	R2*, T1-Vol., 3T	CN, PUT, GP, TH	Automated ROI (3D) analysis; Longitudinal, two serial MRIs	1. Greater annual rates of progression of R2* and volume in the PUT of MSA-P than MSA-C and PD patients. 2. Significant correlation between the R2* and volume changes.
	Lee et al. (64)	21 MSA-P, 18 MSA-C, 22 NC	R2*,T1-Vol., DTI, 3T	CN, PUT, GP, TH, Brainstem, cerebellum	Automated ROI (3D) analysis; Principal component analysis and structural equation modeling to show a model consisting of multiple inter-dependencies	1. No significant correlation between alterations in the R2* of the basal ganglia region and the MRI variables associated with brainstem–cerebellar degeneration.2. Significant correlation between the PUT MD values and the UPDRS and UMSARS scores.
	Barbagallo et al. (65)	16, MSA-P, 13 MSA-C, 26 PD	R2*,T1-Vol., DTI, 3T	SN, CN, PUT	Automated ROI (3D) analysis; Correlation analyses between MRI findings and clinical variables	1. The combination of PUT R2* and MD: >95% discrimination between patients with MSA-P and PD (AUC = 96).2. The UMSARS-II scores correlated with PUT R2* values of the MSA (total) patients, with the PUT MD values of the MSA-P patients, and with the PUT volumes of the MSA-C patients.
	Péran et al. (37)	16 MSA-P, 13 MSA-C, 26 PD, 26 NC	R2*,T1, DTI, 3T	Whole brain	Voxel-based analysis of the gray density, MD, fractional anisotropy, and R2* maps; Unsupervised machine-learning method to classify patients.	1. Several combinations of 2 different markers: >95% discrimination between MSA and PD patients.2. Specific single marker allowed for 95% of discriminant power.3. The unsupervised analysis could regroup individuals according to their clinical diagnosis.

*AUC, area under the curve; CN, caudate nucleus; D, dimensional; DN, dentate nucleus; DTI, diffusion tensor imaging; FDG, Fluorodeoxyglucose; GP, globus pallidus; MD, mean diffusivity; MSA, multiple system atrophy; MSA-P, parkinsonian variant of MSA; MSA-C. cerebellar variant of MSA; MT, magnetization transfer; NC, normal control; PD, Parkinson's disease; PRESTO, principles of echo shifting using a train of observations; PSP, progressive supranuclear palsy; PT, pulvinar thalamus; PUT, putamen; QSM, quantitative susceptibility mapping; RN, red nucleus; ROI, region-of-interest; SI, signal intensity; SN, substantia nigra; SUVR, standardized uptake value; SWI, susceptibility-weighted imaging; T, tesla; TH, thalamus; T1-Vol., T1 volumetry; UMSARS, unified multiple system atrophy rating scale; UPDRS, unified Parkinson's disease rating scale; WM, white matter*.

SWI can provide improved diagnostic accuracy through assessment of the degree and distribution of hypointensity in the putamen using a visual rating scale (Supplementary Figure 1) (41). A pattern of posterolateral putaminal hypointensity with a striking lateral-to-medial gradient on SWI is a highly specific sign of MSA-P (Supplementary Figures 1D–F). However, grading of putaminal hypointensity focused only on the signal intensity without consideration of the distributional pattern fails to differentiate MSA-P from PD (57). Slit-like hypointensity along the lateral margin of the putamen or evenly distributed hypointensity throughout the putamen can be a nonspecific and age-related sign of physiological mineralization (Supplementary Figures 1A–C) (40, 41). Compared with SWI, T2- or T2^*^-weighted images have limited diagnostic value with lower sensitivity (66, 67).

Because of uneven and focal accumulation of iron within the structure of the brain, subregional analyses could have higher diagnostic value than total average iron calculations (33). The posterior and inner subregion of the putamen is the most valuable among four subregional ROIs in differentiating MSA-P from PD. Moreover, high iron deposition percentages can be used to detect smaller increases in iron with higher sensitivity than average SWI phase shifts.

Multimodal MRI can identify specific markers to discriminate patients with PD from patients with MSA with high accuracy (37). The combination of T2^*^ relaxation rates and mean diffusivity (MD) in the putamen achieves discrimination of >95% between patients with MSA-P and PD (65).

### Clinical Correlates

Increased iron content in motor-related subcortical nuclei may be associated with the severity of parkinsonian motor deficits. Nigral iron accumulation in PD correlated with symptoms linked to dopaminergic neurodegeneration (68). A recent study identified a significant correlation between Unified MSA Rating Scale II scores and putaminal R2^*^ values in patients with MSA (65). The phase-shift value of the posterior aspect of the putamen has demonstrated significantly higher asymmetry in MSA-P patients than in PD patients (60). These findings are frequently detected in the contralateral symptomatic side of patients.

In a recent study of correlations between R2^*^ values and the degree of Unified Parkinson's Disease Rating Scale scores in PSP, the burden of iron-related PSP pathologies in the lenticular nucleus was associated with the severity of rigidity, and nigro-striato-pallidal and dentate iron content were associated with the severity of tremors (62). Other studies have failed to show a correlation of iron-sensitive MRI markers with clinical severity scale scores in MSA and PSP (34, 43, 49, 64), but have instead reported that diffusion tensor metrics, such as MD values, are closely correlated with clinical severity (63, 64). Iron-sensitive MRI markers may have less sensitivity to reflect clinical status than diffusion tensor metrics.

Structural MRI abnormalities in MSA are segregated into basal ganglia and brainstem-cerebellar factors (64). Alterations in the R2^*^ of the basal ganglia region are not significantly correlated with the MRI variables associated with brainstem-cerebellar degeneration. In a longitudinal study, a higher rate of iron accumulation was observed in the putamen of MSA-P patients compared to patients with the cerebellar variant of MSA and PD (49). These findings may underlie the stratification of the motor subtype of MSA. Thus, far, no attempt has been made to correlate regional iron deposition with the severity of motor deficits according to the subtypes of PSP.

A recent *post-hoc* analysis demonstrated that MSA-specific MRI abnormalities are associated with more rapid progression and worse overall prognosis (69). In a longitudinal study, greater baseline iron content in the putamen was correlated with smaller volumes at follow-up in MSA patients (49). Similarly, accumulation of iron in the putamen predicts volume loss in healthy older adults (70). Excessive iron accumulation in the putamen may serve as a marker for impending progression of neurodegeneration in MSA.

### Current Limitations and Future Directions

Although recent data suggest a mechanistic link between perturbed iron homeostasis, oxidative stress, and misfolded protein aggregation, whether iron accumulation is a primary pathogenic factor or an epiphenomenon of neuronal degeneration remains to be elucidated. In a single case report, putaminal iron deposition preceded the occurrence of the initial symptoms of MSA-P (71). However, when iron deposition appears during the course of disease is unknown. A longitudinal study on the relationship between multimodal MRI abnormalities at earlier stages of the disease is necessary to identify the spatiotemporal patterns of iron-related neurodegeneration.

High-field MRI developments have advanced the ability to map the distribution of iron *in vivo* (1). However, studies that have investigated the pathological correlates of iron-sensitive MRI measurements are rare. Signal hypointensities observed using T2 or T2^*^ methods are correlated with iron deposits (Fe^3+^) and ferritin in histologic analysis (8, 48, 72). A recent study showed that R2^*^ is significantly associated with nigral α-synuclein. Iron-sensitive MRI may capture the pathological aspects of disorders other than iron (73). Further research is needed to verify the exact underlying pathology of MRI signal alterations.

## Conclusion

A distinctive pattern of iron deposition in MSA and PSP has been described. We highlighted the main factors that can determine region specificity. Disease-specific increases of iron occur in the brain regions mainly affected by underlying disease pathologies. Therefore, *in vivo* MRI mapping of brain iron deposition may serve as an indirect marker of neurodegenerative changes in pathoanatomically relevant sites. Age-related, structure-specific, and sub-structural patterns of iron accumulation should be considered when investigating iron-related neurodegeneration using MRI.

## Author Contributions

All authors listed have made a substantial, direct and intellectual contribution to the work, and approved it for publication.

### Conflict of Interest Statement

The authors declare that the research was conducted in the absence of any commercial or financial relationships that could be construed as a potential conflict of interest.
