# Supplementary material for: Brain Iron Accumulation in Atypical Parkinsonian Syndromes: in vivo MRI Evidences for Distinctive Patterns
Source: Front Neurol. 2019 Feb 12;10:74. doi: 10.3389/fneur.2019.00074 (PMC6379317; doi:10.3389/fneur.2019.00074)
Supplement: Supplementary file 1 [file Data_Sheet_1.PDF]

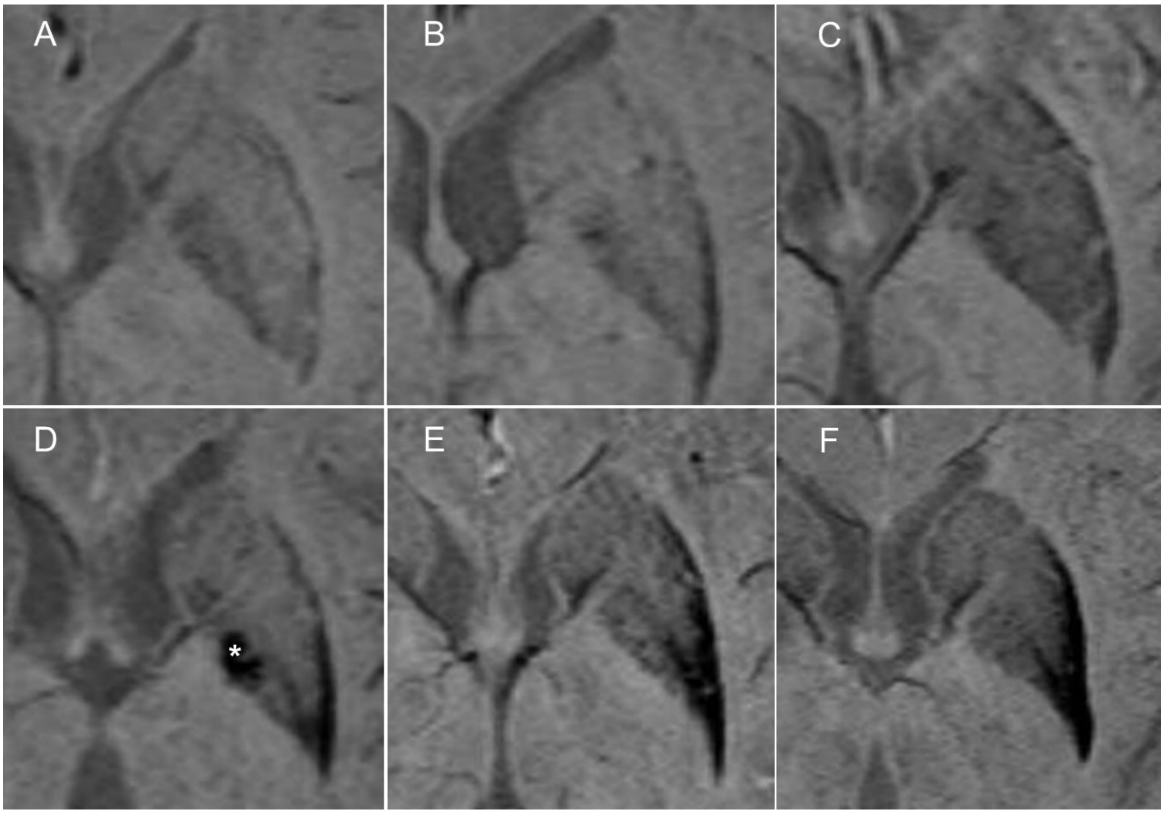

Supplementary Fig. 1 Patterns of putaminal hypointensity on susceptibility weighted imaging for (A–C) normal controls and (D–F) patients with parkinsonian variant of multiple system atrophy. (A) Linear iso-intensity relative to cerebrospinal fluid on the lateral margin; (B) linear hypointensity similar to that of veins on the lateral margin; (C) evenly distributed hypointensity throughout the putamen; (D) marked hypointensity in the posterior putamen with lateral-to-medial gradient extending to the posteromedial putamen; (E, F) marked hypointensity on the most posterior half of the putamen and globus pallidus. *calcification in the globus pallidus. The images were obtained from the Pusan National University Yangsan Hospital using protocols approved by the institutional review board. Written informed consent was obtained from all participants.
